# The impact of poverty alleviation through relocation on loneliness among older adults: the mediating role of social networks and the moderating role of support policies

**DOI:** 10.3389/fpsyg.2026.1748115

**Published:** 2026-02-05

**Authors:** Chuan Li, Ruoxi Zhong, Hao Chen, Yunwei Liu

**Affiliations:** 1 College of Resources and Environment, Xichang University, Xichang, China; 2 School of Economics, Sichuan Agricultural University, Chengdu, China

**Keywords:** loneliness, older adults, poverty alleviation through relocation, social exchange intensity, support policies

## Abstract

**Introduction:**

Poverty Alleviation through Relocation (PAR) aims to break the intergenerational transmission of poverty by improving living environments. Understanding its impact on the psychological well-being of older adults, particularly loneliness, holds significant value for refining post-relocation support policies, enhancing the welfare of older populations, and promoting active aging.

**Methods:**

This study utilizes 2025 field survey data from Sichuan, Yunnan, and Guizhou provinces. The analysis employs an instrumental variable approach to address endogeneity, a mediation effect model to test the role of social exchange intensity, and a moderation effect model to examine the buffering effect of support policies.

**Results:**

Findings indicate that PAR significantly associated with higher overall levels of loneliness among older adults. (*β* = 0.568, *p* < 0.01). However, PAR exerts differential effects on distinct dimensions of loneliness: it significantly aggravates emotional loneliness (*β* = 3.592, *p* < 0.01) while alleviating social loneliness (*β* = −1.486, p < 0.01). Heterogeneity analysis further reveals that the vulnerability to loneliness is more prominent among older adults in centralized resettlement communities and those in the older-old age group. Mechanism analysis suggests that social exchange intensity mediates the effect of PAR on loneliness. Support policies effectively mitigate the negative impact of PAR on emotional loneliness.

**Discussion:**

This study reveals that while PAR improves material conditions, it may simultaneously create structural risks for the psychological well-being of older adults by disrupting social networks and weakening emotional support. The study recommends building differentiated psychological support systems, strengthening mechanisms for social network reconstruction, improving targeted support for vulnerable groups, and integrating mental health services into community governance. These measures can systematically enhance the psychological well-being of older adults in PAR communities.

## 1 Introduction

Against the dual backdrop of an accelerating population aging process and increasing societal mobility, the psychological wellbeing of older adults, particularly their loneliness, has emerged as a significant public health and social psychological wellbeing issue that demands attention (Masi et al., 2011; Li et al., 2019). Loneliness extends far beyond a simple negative emotion; substantial evidence associates it with a series of serious physical and psychological problems (Mann et al., 2017). Within the unique context of Chinese family culture and rapid social transformation, the decline of traditional multi-generational households and the prevalence of the “empty nest” phenomenon in rural areas are impacting and reshaping the traditional social support networks that older adults rely on. These changes make issues of psychological adaptation and loneliness among older adults increasingly prominent.

As a core initiative of China’s Targeted Poverty Alleviation strategy, Poverty Alleviation through Relocation (PAR) brings developmental opportunities to the rural poor by fundamentally disrupting the intergenerational transmission of poverty through changes in living environments (Wang et al., 2023). However, alongside the focus on the material improvements PAR provides, such as economic gains and better housing conditions, academia must give equal attention to the long-term impact of PAR on the social psychological wellbeing of migrants. The psychological impact on older adults, a particularly vulnerable group, requires urgent investigation. Deeply influenced by a traditional cultural attachment to their native land, older adults in China have social networks, life values, and a sense of meaning highly embedded in the geographical and social spaces of their original communities (Chen and Wang, 2023; Chen and Sui, 2013). The relocation process, while improving material living conditions, may also rupture their original social capital, cause a loss of place attachment, and dismantle familiar rural support systems. These disruptions can potentially trigger or exacerbate feelings of loneliness (Wu and Penning, 2015; Laboy et al., 2009).

Therefore, this study employs field survey data from Poverty Alleviation through Relocation (PAR) to systematically address the following core questions: First, does PAR alleviate or aggravate loneliness among older adults? Second, does this impact demonstrate heterogeneity across the two dimensions of emotional loneliness and social loneliness? Third, how does PAR influence loneliness among older adults through the mechanism of social networks and the utilization of support policies? Fourth, do these effects differ based on resettlement modes (centralized/dispersed) and age groups (younger-old/older-old)? Compared with existing research, this study deconstructs loneliness into “emotional” and “social” dimensions. This approach provides a more refined analytical framework for investigating the mental health of older adults in the PAR context. The framework helps reveal the complexity and paradoxical nature of how PAR influences the psychological wellbeing of older adults, thereby offering a scientific basis for improving targeted support services and mental health care systems for this population.

## 2 Literature review

Loneliness is a widespread subjective psychological experience recognized as a core indicator affecting the quality of life and psychological wellbeing of older adults in aging research (Jose et al., 2014; Buz and Pérez-Arechaederra, 2014; Oshagan and Allen, 1992). Scholarly understanding of its nature has evolved from a unidimensional concept to a multidimensional construct. Classic theoretical frameworks on the dimensions of loneliness hold pioneering significance for explicitly distinguishing between emotional loneliness, which stems from the absence of close attachment relationships, and social loneliness, which arises from a lack of social network membership (Suedfeld, 1977; Weiner, 1975; Malerstein, 1976). This distinction possesses strong explanatory power for understanding major turning points in the life course (Safro et al., 1996; Jang and Tang, 2022). However, applying this multidimensional loneliness theoretical framework to research on older adults undergoing PAR remains an underexplored area. Currently, few specialized studies focus specifically on the loneliness of older adults in PAR settings, and related discussions concentrating on the mental health of this migrant group are also relatively limited. Most existing studies adhere to a unidimensional measurement of loneliness (Cao et al., 2024; Zhang, 2023) or subsume it within broader conceptual frameworks such as social adaptation or psychological integration for examination (Chen and Zhang, 2025; Yuan et al., 2025; Liu, 2023; Wang and Chen, 2016). While these existing studies offer significant reference value, they may obscure the complex and even contradictory impact mechanisms that PAR exerts on the psychological world of older adults. For instance, well-equipped resettlement areas might alleviate social loneliness by increasing social opportunities. Conversely, separation from adult children, often caused by limited apartment sizes or intergenerational lifestyle differences, could significantly aggravate their emotional loneliness.

Regarding the impact of migration and resettlement on mental health, academia holds two seemingly opposing theoretical perspectives, namely the “Facilitation Thesis” and the “Inhibition Thesis.” These perspectives provide competing theoretical hypotheses for analyzing the impact of Poverty Alleviation through Relocation (PAR) on loneliness among older adults. The “Facilitation Thesis” primarily argues from the perspectives of resource accessibility and environmental improvement. Proponents argue that PAR enables older adults to move away from their original residences characterized by harsh natural conditions and poor infrastructure. This transition provides them with safer and more comfortable housing, more accessible medical services, and more abundant community public spaces (Rich Madsen et al., 2016; Lee, 2010). These material environmental improvements not only directly enhance living convenience but also create more opportunities for social interaction for older adults. Consequently, PAR may effectively alleviate the social loneliness stemming from physical isolation and social exclusion (Li et al., 2023). Furthermore, the dispersed resettlement mode helps to maintain the stability of their social capital by partially preserving original neighborhood relations and production lifestyles (Zhang et al., 2022; Zhang and Shi, 2021).

The “Inhibition Thesis,” in contrast, focuses on social network disruption and psychological trauma. This perspective emphasizes that for older adults in China, who are deeply embedded in a native soil society, PAR constitutes not merely a geographical relocation, but a rupture of social relationships, cultural identity, and lifestyle (Serra et al., 2022). The compulsory spatial displacement leads to the disintegration of their long-established, strong-tie networks based on geography and kinship, thereby triggering profound emotional loneliness (Berry, 1997; Shekelle et al., 2024). Simultaneously, the transition from the familiar role of an agricultural producer to a “marginalized” individual in an urban community creates a strong sense of skill obsolescence and identity crisis. This role loss, when uncompensated by new sources of value, further exacerbates feelings of loneliness (Sreelekha and Sia, 2022; Mehra et al., 2024). Furthermore, the rigid increase in living costs may impose persistent financial pressure on economically constrained older adults, indirectly affecting their psychological wellbeing (Shi and Yu, 2023). However, the impact of PAR on loneliness among older adults is not a simple linear relationship. It is rather a complex process interwoven with both positive and negative effects, the final outcome of which likely depends on the interplay of these two forces and the operation of a series of mediating and moderating mechanisms.

A systematic review of the existing literature reveals that, although the multidimensional theoretical framework of loneliness has found application in the field of mental health among older adults, its utilization in research on older adults within the Chinese Poverty Alleviation through Relocation (PAR) context remains markedly insufficient. Current research has not yet sufficiently revealed the potential differential impacts of PAR on emotional loneliness versus social loneliness. These research gaps not only limit the in-depth understanding of the psychological adaptation processes of older adults but also hinder the precise formulation and implementation of subsequent support policies.

## 3 Theoretical analysis and research hypotheses

Poverty Alleviation through Relocation (PAR) brings fundamental improvements to the physical living environment for older adults, but it also signifies a drastic reconstruction of the long-established socio-ecological environment to which they are attached. Grounded in the aforementioned multidimensional loneliness theoretical framework, this study moves beyond a focus on loneliness to deconstruct loneliness among older adults into two dimensions: emotional loneliness and social loneliness. This approach aims to systematically reveal the compound impact of PAR on their psychological wellbeing. The rationale for this deconstruction is twofold. On one hand, PAR may alleviate social loneliness caused by a lack of social resources in their original residences by providing better public facilities, richer community activities, and more frequent opportunities for neighborly contact. On the other hand, for older adults with a strong attachment to their native land, the compulsory nature of PAR severs their emotional bonds with the original community, potentially causing a rupture of deep-seated place attachment. Concurrently, the common phenomenon of intergenerational separation following PAR may directly weaken their most crucial source of emotional support, thereby aggravating emotional loneliness. Given the high dependence of older adults in rural China on social networks and family support, coupled with their relatively limited capacity for environmental adaptation, we anticipate that the negative impacts of social network disruption and weakened emotional support caused by PAR are likely to outweigh the initial positive effects of improved living conditions. Therefore, this study proposes the following hypotheses:

*H1*: PAR aggravates loneliness among older adults.

*H2*: PAR aggravates emotional loneliness among older adults.

*H3*: PAR alleviates social loneliness among older adults.

Social capital can be understood as the sum of actual or potential resources embedded in social relationships that provide support to individuals (Malerstein, 1976; Aguiar, 2002). PAR not only changes residential space but also reconstructs the social relational structure on which older adults rely: the strong-tie network of “bonding social capital” centered on kinship and locality may be weakened, while new weak-tie networks of “bridging social capital” among community neighbors gradually emerge (Nyqvist et al., 2013; Rautiainen et al., 2024). In essence, individuals’ social connectedness is sustained through “ongoing interactions of resource exchange,” and the spatial displacement brought about by PAR directly disrupts the geographic and social foundations of exchange that depend on physical proximity and long-term trust accumulation. This imbalance in the exchange structure constitutes the core mechanism through which PAR exerts differential effects on various dimensions of loneliness, and it also implies that social exchange is a key pathway linking PAR to loneliness. Based on the above theoretical analysis, this study proposes the following research hypotheses:

*H4*: Social exchange intensity mediates the relationship between relocation and loneliness among older adults.

External support resources can effectively mitigate the negative impact of stressful life events on individual psychological wellbeing. As a formal, institutionalized external support led by the government, support policies compensate for the weakened informal support networks resulting from PAR by providing services such as daily care, regular visits, and emotional support. This compensation directly buffers the impact of PAR on emotional loneliness (Sharma and Bista, 2025). By organizing community activities, creating communication opportunities, and improving public spaces, support policies help construct new social interaction platforms for older adults. This process not only facilitates the establishment of new social connections but also actively promotes the alleviation of social loneliness. Therefore, this study proposes the following research hypothesis:

*H5*: Support policies play a positive moderating role between PAR and loneliness among older adults.

**Figure 1:**
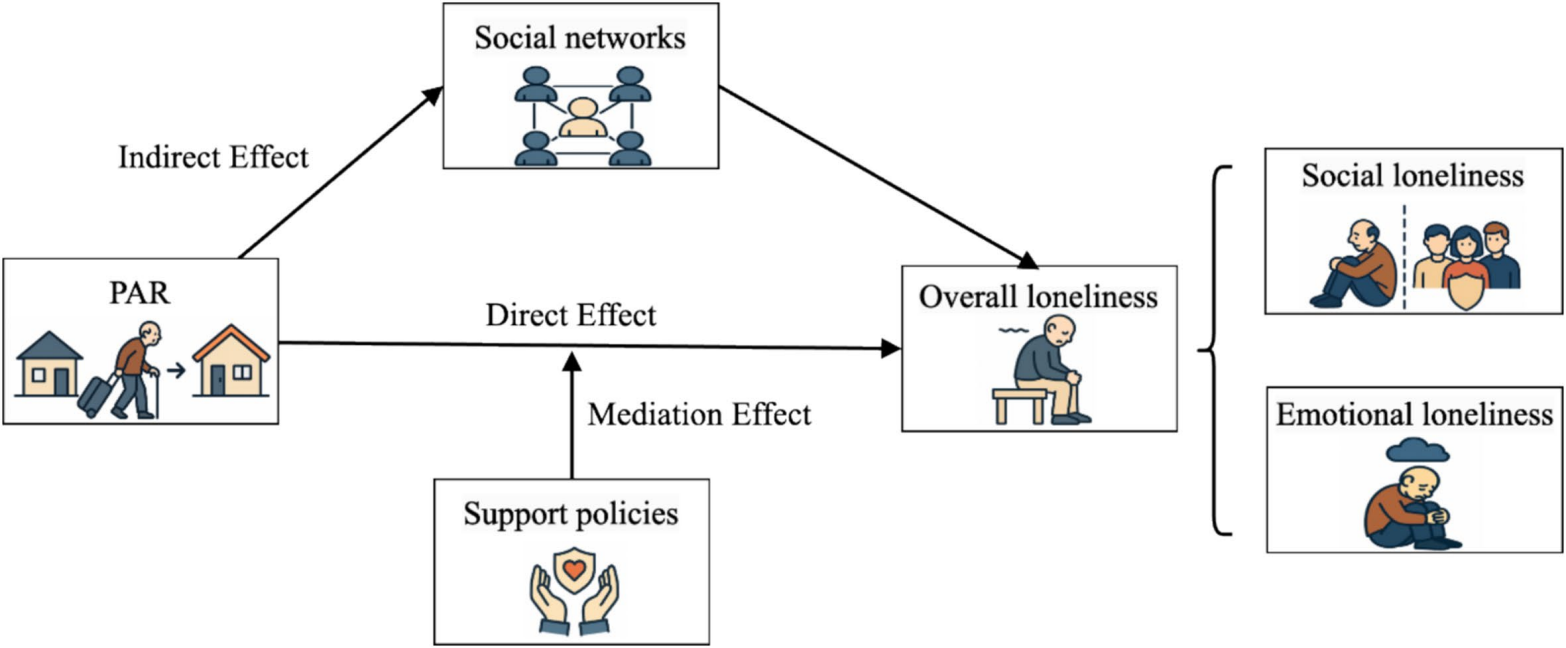
Mechanism of action diagram.

Figure 1 illustrates the mechanism of action diagram described in this paper.

## 4 Data sources, variable selection, and research methods

### 4.1 Data sources

This study utilizes microdata obtained from a field survey on the social adaptation and psychological wellbeing of older adults undergoing PAR. The survey was conducted by the research team between February and March, and again from June to August 2025. It covered key PAR implementation areas in Sichuan, Yunnan, and Guizhou provinces, China, including core zones of contiguous impoverished areas and surrounding regions, ensuring good representativeness. The survey employed a stratified random sampling strategy, using PAR resettlement communities as the primary sampling units and selecting older adults as respondents. Specifically, in key PAR implementation areas in Sichuan, Yunnan, and Guizhou Provinces, the sample was stratified by resettlement modality and regional type. Resettlement communities were then taken as the sampling units, from which relocated older adults were randomly selected to form the treatment group (PAR = 1). To construct a comparable control group (PAR = 0) and strengthen identification of the effect of relocation, the research team also selected, near the original places of residence corresponding to the resettlement communities, natural villages that had not undergone full relocation or where PAR had not been implemented, but were similar in geographic location, economic conditions, and sociocultural context. Eligible non-relocated older adults were randomly drawn from these villages as the comparison group. This design ensured that the non-relocated sample largely came from areas comparable to the treatment group in terms of living background, policy environment, and social structure, thereby improving the comparability of loneliness between relocated and non-relocated older adults to some extent. Although we reduced selection bias by matching on geographic and socioeconomic conditions, relocation decisions may still be influenced by unobserved factors such as household risk preferences and heterogeneity in local policy implementation. To address this concern, we included a rich set of household- and individual-level covariates in the econometric models and employed an instrumental-variable approach to mitigate endogeneity and the resulting estimation bias.

It should be noted that the Poverty Alleviation Relocation (PAR) examined in this study refers to a one-off, large-scale resettlement program that was centrally planned and implemented by the government during China’s targeted poverty alleviation campaign, under the framework of the National 13th Five-Year Plan for Poverty Alleviation through Relocation. In our sample, the relocation took place in 2020. This relocation was clearly irreversible: housing at the original sites was demolished, and homestead land was reclaimed for cultivation or converted to forest. As a result, relocated households faced substantial physical and economic constraints that made long-term return to their original locations highly unlikely. Fieldwork further confirmed that no return migration occurred. The survey distributed 1,100 questionnaires and retrieved 1,094. Following rigorous data cleaning, which involved removing samples with missing key variables or logical inconsistencies, 1,081 valid questionnaires remained. The effective response rate was 98.27%. The final valid sample provides a solid data foundation for subsequent empirical analysis, offering a sound representation of the study population.

### 4.2 Variable selection

#### 4.2.1 Explained variable

Social network changes and adaptation pressures induced by relocation may significantly affect loneliness among older adults. In this study, loneliness is measured along three dimensions—overall loneliness, emotional loneliness, and social loneliness (Buz and Pérez-Arechaederra, 2014; Oshagan and Allen, 1992). The use of single-item measures is primarily motivated by multiple considerations regarding respondent characteristics and field feasibility. Our sample consists of older adults participating in rural PAR in deeply impoverished areas of Southwest China. Most respondents are illiterate or semi-literate and primarily communicate in local dialects in daily life. Accordingly, the survey relied mainly on trained interviewers who were fluent in both local dialects and Mandarin to conduct face-to-face interviews. Under such conditions, multi-item scales face fundamental challenges, including respondents’ inability to read, increased cognitive burden from complex wording, and potential ambiguities arising from dialect translation of culturally loaded concepts. In this context, single questions are more likely to capture older adults’ authentic subjective feelings and lived experiences of loneliness. A growing body of empirical research on rural older adults in China has adopted single-item measures of loneliness under similar practical constraints and has been recognized in the literature (Li et al., 2019; Tang et al., 2022; Zhou et al., 2020).

This study draws on Weiss’s classic theoretical typology of loneliness (Weiss, 1973) and conceptualizes loneliness as comprising two dimensions—emotional loneliness and social loneliness. Each dimension is measured using a single item with relatively high face validity:

Overall loneliness is measured by the statement: “I often feel very lonely.” This item directly captures the respondent’s overall subjective experience of loneliness and has been widely used as a valid proxy for loneliness in studies of older adults (Li et al., 2019; Jose et al., 2014). Its brevity facilitates comprehension among respondents with limited educational attainment and helps them report their feelings more accurately.

Emotional loneliness is measured by: “I often feel that I lack companionship from family members.” Weiss argues that emotional loneliness stems from the absence of close attachment relationships (Weiss, 1973). In the context of Chinese family culture, companionship from relatives—especially adult children—constitutes a core source of emotional support for older adults. Therefore, perceived “lack of family companionship” serves as a direct and effective indicator of emotional loneliness (Chen and Wang, 2023; Jang and Tang, 2022).

Social loneliness is measured by: “I rarely communicate with other community residents.” Weiss defines social loneliness as the perceived absence of membership in a social network, namely, a lack of belonging to a social group (Weiss, 1973). After relocation, the resettlement community becomes the primary arena for older adults’ social activities. The frequency of communication with community residents provides a direct behavioral manifestation of whether social ties are being established in the new community, the extent of social participation, and the absence of a sense of belonging. Although this item directly measures interaction frequency, prior research commonly suggests that, within community settings, the frequency of social interaction is highly correlated with individuals’ perceived deficiency in social connectedness (Zhang et al., 2022; Nyqvist et al., 2013). For relocated older adults, limited communication often directly reflects weak social networks and difficulties in community integration, thereby pointing to the lived experience of social loneliness.

In this study, overall loneliness, emotional loneliness, and social loneliness are all measured on a five-point Likert scale, ranging from 1 (strongly disagree) to 5 (strongly agree), with higher scores indicating stronger loneliness. Given that the distributions of these three indicators in the sample approximate continuity, and that Likert-scale values are often treated in psychological and sociological research as approximations of an underlying continuous latent construct, we treat them as continuous variables in the baseline regressions and estimate linear models. This differentiated measurement strategy enables the study to examine the complex effects of relocation on older adults’ loneliness more comprehensively and in greater depth from three complementary perspectives: overall feelings, emotional connectedness, and social integration.

#### 4.2.2 Core explanatory variable

The key explanatory variable in this study is whether an older adult settled in a resettlement community as a result of the Poverty Alleviation through Relocation (PAR) policy. This is a binary indicator: it equals 1 (treatment group) if the respondent moved from their original place of residence and settled in a centrally built, government-constructed resettlement site under the policy; otherwise, it equals 0 (control group). To ensure measurement accuracy, the research team verified the relocation year and main places of origin for each resettlement community by consulting administrative management records and interviewing community staff. In addition, the questionnaire included two items—“In which year did you move to the resettlement community through the PAR program?” and “Have you settled in this resettlement community?”—to further confirm respondents’ relocation experience and whether they had indeed settled in the resettlement community. In our sample, the relocation occurred in 2020, implying no heterogeneity in relocation duration. Moreover, relocation predates both the mediator (household gift expenditures in 2024) and the outcome (loneliness measured in 2025). This ensures a clear temporal ordering of variables and provides a chronological basis for the subsequent mechanism analysis.

#### 4.2.3 Instrumental variable

To address endogeneity concerns, this study uses geological disaster risk in the respondent’s original place of residence as an instrumental variable. Specifically, the instrument is defined based on whether the respondent’s original village was classified as a high-risk geological hazard area in the government’s PAR planning. If a village was identified as a high-risk site for hazards such as landslides or debris flows, the variable is coded as 1; otherwise, it is coded as 0. The relevant information is drawn from local government documents on geological hazard risk screening and village-level risk grading conducted prior to the implementation of PAR. The literature also generally recognizes geological hazard risk as an important technical basis for determining eligible relocation targets and delineating resettlement coverage (Wang, 2018; Xu et al., 2025).

The validity of this instrumental variable can be justified on two grounds. First, in terms of relevance, geological disaster risk in the original place of residence is an important consideration for the government when deciding whether, and with what priority, to implement relocation across villages. The higher the assessed risk level, the more likely a village and its households are to be included in the relocation plan (Zhu and Yu, 2021; Zhang and Guo, 2019). Second, with respect to the exclusion restriction, this indicator captures long-term and relatively stable exposure to natural geographic risk, rather than short-term, shock-driven disaster events (Wang et al., 2018). Approximately 5 years after relocation, the relocated older adults in our sample have largely detached from their original high-risk environments. Loneliness is measured in the new resettlement communities, which are physically decoupled from the original natural risk conditions. It is therefore reasonable to assume that any effect of the original geological risk on loneliness operates primarily through participation in the PAR policy, rather than through a direct channel.

#### 4.2.4 Mediating variable and moderating variable

Social exchange intensity is regarded as an important structural resource for buffering loneliness and a key pathway through which PAR affects older adults’ mental health. Compared with psychological resources, which place greater emphasis on individuals’ subjective feelings and internal states, and social capital, which highlights actual or potential resources embedded in social relationships, social exchange focuses on the ongoing process of reciprocal resource exchange and interaction between individuals. It serves as the concrete behavioral vehicle through which social capital is formed, maintained, and activated, and it constitutes a foundational channel through which perceived psychological support is generated (Berger et al., 1972; Gao et al., 2025; Wang et al., 2025). In the context of PAR, spatial displacement directly disrupts older adults’ pre-existing exchange networks grounded in locality and kinship. This disruption may lead not only to a loss of social capital but also to a weakening of psychological resources such as a sense of belonging and security (Ye and Wang, 2022; Wang and Jiang, 2020; Wu and Niu, 2018). Accordingly, examining changes in social exchange intensity allows us to capture the most immediate and fundamental shifts in social relationships induced by relocation, thereby illuminating the key behavioral and relational mechanisms through which relocation influences mental health.

Social exchange intensity is expected to serve as a key mediating pathway between relocation and loneliness for the following reasons. PAR disrupts older adults’ pre-existing exchange networks, which may directly reduce the frequency of social interactions and reciprocal support, thereby exacerbating loneliness. At the same time, rebuilding social exchange networks in the new environment is also a process through which older adults obtain new forms of support and re-establish a sense of belonging; if successful, it may alleviate loneliness. Therefore, social exchange intensity constitutes a core transmission channel through which PAR affects older adults’ psychological adaptation.

In the context of China’s rural (acquaintance-based) society, gift-giving exchanges (monetary gifts) constitute a core ritualized carrier of social exchange. They not only reflect the strength with which strong ties among relatives are maintained but also capture the closeness of relationships among neighbors and community members. As such, gift exchanges can serve as an integrated indicator of both the existence of social connectedness and the frequency of interaction. They represent an important mechanism through which households sustain social interactions, fulfill reciprocity norms, and participate in social exchange networks, and their magnitude largely reflects a household’s interaction frequency and level of embeddedness in these networks (Wu and Niu, 2018; Lei, 2024; Liu, 2024). In other words, higher gift expenditures are typically associated with greater intensity of participation in social exchange and more active social interactions. Therefore, this study uses household gift expenditures as a measure of social exchange intensity, aiming to empirically test the mediating mechanism proposed above.

As an institutionalized support mechanism led by the government, support policies provide essential resources and security for older adults undergoing PAR. This study incorporates support policies into the analytical framework to examine how this external support factor exerts a moderating effect between PAR and loneliness among older adults.

#### 4.2.5 Control variables

Individual characteristics serve as a key element for assessing personal psychological vulnerability, while family capital constitutes a core resource for resisting psychological risks. The analysis therefore controls for older adults’ individual characteristics, family characteristics, and PAR characteristics separately. Regional dummy variables capture unobserved differences across provinces in economic development, policy implementation, and cultural practices, ensuring that the estimation results are robust to regional heterogeneity. Table 1 details the specific definitions of all variables and presents the descriptive statistical analysis.

**Table 1:** Variable definitions and descriptive statistics.

Variable category	Variable	Definition	Mean	Std. dev.
Explained variable	Overall loneliness	Self-assessment of loneliness (five-point scale)	2.67	1.58
	Emotional loneliness	Assessment of need for family companionship (five-point scale)	3.45	1.49
	Social loneliness	Assessment of community integration and communication (five-point scale)	2.77	1.55
Core explanatory variable	PAR	Whether participated in PAR (Yes = 1; No = 0)	0.66	0.48
Individual characteristics	Political	Whether a Communist Party member (Yes = 1; No = 0)	0.03	0.18
	Education	Educational attainment (1 = Primary school and below; 2 = Junior high; 3 = Senior high and above)	1.09	0.37
Household characteristics	Laborer	Number of laborers in the household	2.87	1.72
	H_ducation	Number of household members with a senior high school education or above	0.37	0.62
	Health	Number of healthy household members	2.91	1.91
	P_land area	Logarithm of (operated land area / total household members)	1.68	1.26
	P_fixed assets	Logarithm of (total household fixed assets / total household members)	7.55	1.10
	Saving	Logarithm of total household savings in 2024	7.14	4.71
	Loans	Whether the household has access to loans (Yes = 1; No = 0)	0.53	0.50
	Borrowing	Whether the household has access to borrowing (Yes = 1; No = 0)	0.63	0.48
Relocation characteristics	Poverty	Whether lifted out of poverty at the time of PAR (Yes = 1; No = 0)	0.03	0.17
	Distance	Logarithm of the distance between the relocation site and the original residence	2.71	0.75
	Scale	Number of people relocated from the original village	3.81	0.33
Regional dummy variables	Region	1 = Sichuan; 2 = Yunnan; 3 = Guizhou	—	—
Instrumental variable	Risk	Whether the original place of residence is a high geological hazard risk area (Yes = 1; No = 0).	0.57	0.49
Mediating variable	social exchange intensity	Logarithm of total household gift expenditure in 2024	8.23	1.78
Moderating variable	Support policies	Whether received various support policies (Yes = 1; No = 0)	0.44	0.50

### 4.3 Research methods

#### 4.3.1 Baseline regression

The three outcomes measure overall loneliness, emotional loneliness, and social loneliness—are each recorded on a five-point Likert scale and are therefore *ordered discrete* variables. To fully respect their ordinal nature and avoid potential bias that may arise from treating adjacent categories as equally spaced, we employ an ordered Probit model in the baseline regressions. The model is specified as follows:


$${\text{Loneliness}}_{i2}=\beta_{0}+\beta_{1}{\text{Relocation}}_{i}+\gamma_{i}Z_{i}+\delta \text{County}+\varepsilon_{i}$$ (1)

In the Equation 1, ${\text{Loneliness}}_{i2}$ represents the level of loneliness among older adults, ${\text{Relocation}}_{i}$ denotes participation in PAR, $Z_{i}$ represents the vector of control variables, County denotes regional dummy variables, $\varepsilon_{i}$ is the random error term, and $\beta_{0}$ , $\beta_{1}$ , and $\gamma_{i}$ are the estimated coefficients.

#### 4.3.2 Instrumental variables model

Building upon the baseline regression model, this study employs the following model to address potential endogeneity issues:


$$\hat{{\text{Relocation}}_{i}}=\partial_{0}+\partial_{1}\mathrm{IV}+\gamma_{i}Z_{i}+\delta \text{County}+\varepsilon_{i}$$ (2)

$${\text{Loneliness}}_{i}=\beta_{0}+\beta_{1}\hat{{\text{Relocation}}_{i}}+\gamma_{i}Z_{i}+\delta \text{County}+\varepsilon_{i}$$ (3)

In the Equations 2, 3, *IV* represents the instrumental variable. In the first stage, the instrumental variable isolates the exogenous variation of the endogenous variable to generate the fitted value $\hat{{\text{Relocation}}_{i}}$ . In the second stage of the model, this fitted value $\hat{{\text{Relocation}}_{i}}$ is incorporated into the model to estimate the remaining coefficients. Where ${\hat{\text{Relocation}}}_{i}$ is the fitted value, IV is the instrumental variable, ${\text{Loneliness}}_{i}$ denotes older adults’ loneliness, ${\text{Relocation}}_{i}$ indicates whether the individual experienced PAR relocation, $Z_{i}$ is a vector of control variables, County denotes regional dummy variables, $\varepsilon_{i}$ is the error term, and β , θ , and γ are parameters to be estimated.

#### 4.3.3 Mediation effect model

Following the established procedure for testing mediation effects (Wen et al., 2022; Wen and Ye, 2014), this study constructs the following models to examine the mediating role of social capital:


$$\begin{array}{l} {\text{Loneliness}}_{i}=\delta_{0}+\delta_{1}{\text{Relocation}}_{i}+\delta_{2}{\text{Mediator}}_{i}\\ +\delta_{3}Z_{i}+\delta_{4}{\text{County}}_{i}+\varepsilon_{i} \end{array}$$ (4)

In the Equation 4, $\text{Lonelines}s_{i}$ represents the loneliness of older adults, $\text{Mediato}r_{i}$ denotes the mediating variable, $\text{Count}y_{i}$ indicates regional dummy variables, $\varepsilon_{i}$ is the random error term, and $\delta_{i}$ are the estimated coefficients. The meanings of other characters are consistent with Equation 1.

#### 4.3.4 Moderation effect model

This study establishes the following model to examine the moderating effect of support policies:


$$\begin{array}{l} {\text{Loneliness}}_{i}=\gamma_{0}+\gamma_{1}{\text{Relocation}}_{i}+\gamma_{2}{\text{Policy}}_{i}+\gamma_{3}{\text{Relocation}}_{i}\\ {}*{\text{Policy}}_{i}+\gamma_{i}Z_{i}+\gamma_{5}\text{County}+\varepsilon_{i} \end{array}$$ (5)

In the Equation 5, where Loneliness, denotes the older adult’s loneliness ${\text{Relocation}}_{i}$ indicates whether the household participated in PAR relocation, $Z_{i}$ is a vector of control variables, ${\text{Policy}}_{i}$ represents post-relocation follow-up support policies, County denotes regional dummy variables, $\varepsilon_{i}$ s the random error term, and β , γ , and δ are the coefficients to be estimated. In this study, the interaction term between household relocation and post-relocation support policies is used to test the moderating effect of follow-up support policies on the relationship between household relocation and loneliness among relocated older adults.

Given the different types of dependent variables, this study adopts model specifications tailored to each analytical context. First, overall loneliness, emotional loneliness, and social loneliness are all five-category ordinal variables; accordingly, the baseline regressions are estimated using an ordered Probit model to examine the association between PAR relocation and loneliness. Second, to address potential endogeneity, we employ an instrumental-variable Probit (IV-Probit) model, using geological disaster risk as the instrumental variable, to identify the robust direction of the effect of relocation on loneliness. Third, for the mechanism analysis and the moderating-effect analysis, we continue to use ordered Probit models based on the ordinal nature of the variables, and we also report corresponding alternative models to assess the robustness of the findings.

## 5 Empirical results and analysis

### 5.1 Baseline regression results

Because the dependent variable is a five-category ordered measure of loneliness, this study estimates an ordered Probit model. The coefficients are primarily interpreted in terms of the direction and statistical significance of the effects. Table 2 reports the regression results of PAR on loneliness among older adults. Columns (1) and (2) use overall loneliness as the dependent variable. Columns (3) to (4) and columns (5) to (6) use emotional loneliness and social loneliness as the dependent variables, respectively. Regarding overall loneliness, the coefficient for PAR was 2.833 and highly significant at the 1% level when no control variables were included. After introducing regional fixed effects and all control variables, the coefficient for PAR decreased to 0.568, but its positive significance remained at the 1% level. This indicates that although regional differences and individual household characteristics partially explain the impact of PAR on loneliness, PAR remains a significant factor aggravating loneliness among older adults. Hypothesis H1 is supported.

**Table 2:** Baseline regression results of the impact of PAR on the loneliness of older adults.

Variable	(1)	(2)	(3)	(4)	(5)	(6)
	Overall loneliness	Overall loneliness	Emotional loneliness	Emotional loneliness	Social loneliness	Social loneliness
PAR	2.833^***^	0.568^***^	3.926^***^	3.592^***^	−2.845^***^	−1.486^***^
	(0.119)	(0.183)	(0.222)	(0.351)	(0.103)	(0.152)
Individual characteristics	NO	YES	NO	YES	NO	YES
Household characteristics	NO	YES	NO	YES	NO	YES
Relocation characteristics	NO	YES	NO	YES	NO	YES
Region	NO	YES	NO	YES	NO	YES
Log likelihood	−1181.55	−655.55	−1106.94	−616.93	−1184.29	−755.49
Pseudo-*R*^2^	0.263	0.591	0.343	0.634	0.292	0.548
*N*	1,081	1,081	1,081	1,081	1,081	1,081

*, **, *** Indicate significance at the 10, 5, and 1% levels, respectively. The values in parentheses represent standard errors.

The results further reveal the impact of PAR on different dimensions of loneliness. For emotional loneliness, the coefficient for PAR was consistently positive and highly significant (*β* = 3.592, *p* < 0.01). This suggests that PAR significantly aggravates loneliness stemming from the lack of intimate emotional connections among older adults. In sharp contrast, for social loneliness, the coefficient for PAR was significantly negative (*β* = −1.486, *p* < 0.01). This implies that PAR alleviates loneliness arising from the lack of social networks to some extent. Hypotheses H2 and H3 are supported.

In summary, PAR aggravates overall loneliness among older adults, and this effect exhibits profound dimensional differences. PAR significantly aggravates emotional loneliness while producing a positive effect on alleviating social loneliness. This finding underscores the importance of deconstructing loneliness among older adults. It demonstrates that a simplistic discussion of PAR as “good” or “bad” would mask its complex psychosocial effects.

### 5.2 Heterogeneity test

Table 3 presents the regression results grouped by PAR resettlement modes. In the centralized resettlement group, PAR demonstrates significant effects on overall loneliness, emotional loneliness, and social loneliness. The coefficient for overall loneliness is 0.407 (*p* < 0.01), for emotional loneliness is 3.593 (*p* < 0.01), and for social loneliness is −1.657 (*p* < 0.01). These results indicate that under centralized resettlement conditions, PAR significantly aggravates overall loneliness and emotional loneliness among older adults, while simultaneously alleviating social loneliness. In contrast, in the dispersed resettlement group, PAR’s impact on loneliness among older adults does not show statistical significance in any dimension. This result forms a sharp contrast with the centralized resettlement group, suggesting that resettlement mode plays a differential role in how PAR affects loneliness. The dispersed mode, due to its proximity to original residences, largely preserves pre-existing social relations and production structures, resulting in no significant impact of PAR on loneliness.

**Table 3:** Heterogeneity analysis: grouped regression results by resettlement mode.

Variable	Centralized resettlement			Dispersed resettlement		
	Overall loneliness	Emotional loneliness	Social loneliness	Overall loneliness	Emotional loneliness	Social loneliness
PAR	0.407^***^	3.593^***^	−1.657^***^	6.622	7.010	−0.797
	(0.197)	(0.360)	(0.166)	(0.445)	(0.979)	(0.528)
Controls	YES	YES	YES	YES	YES	YES
Region	YES	YES	YES	YES	YES	YES
Loglikelihood	−437.38	−472.60	−571.12	−201.86	−137.428	−162.88
Pseudo-*R*^2^	0.607	0.643	0.549	0.497	0.530	0.488
*N*	813	813	813	268	268	268

*, **, *** Indicate significance at the 10, 5, and 1% levels, respectively. The values in parentheses represent standard errors.

Centralized resettlement sites typically accommodate thousands or even tens of thousands of people, with an average travel time of 5–6 h from the original residences. This large-scale, long-distance migration substantially weakens the kinship networks based on blood and geographical ties that older adults rely on. Furthermore, as PAR implementation uses the household as the unit, family separation becomes common, forcing older adults apart from some of their children and relatives. This severs daily emotional support and significantly aggravates their emotional loneliness. On the other hand, the high-density community environment created by large-scale centralized resettlement, along with various community activities organized by the government to promote integration, creates conditions for older adults to expand new community networks. These factors increase their opportunities for social interaction with peers and neighbors, thereby alleviating the social loneliness caused by the loss of original community connections to some extent.

Significant differences exist in physiological function, social roles, and psychological adaptation abilities among older adults across different age stages. This study divides the respondents into two groups: younger-old adults (60–70 years) and older-old adults (over 70 years) to examine age heterogeneity. Age 70 is commonly regarded as a key threshold at which physiological functioning declines more markedly and the risk of chronic diseases rises substantially; at this stage, older adults’ capacity for environmental adaptation and social participation also begins to deteriorate more noticeably. In rural China, individuals aged 70 and above typically withdraw from agricultural labor and increasingly assume care-dependent roles within the household, accompanied by important changes in their patterns of social participation and emotional reliance. Therefore, this cutoff is not only widely accepted in academic research but also better aligns with China’s local policy context, facilitating closer linkage between empirical findings and policy practice. Table 4 presents the regression results grouped by age. Within the younger-old adult group, the coefficient for PAR on overall loneliness is 0.932 (*p* < 0.01), and the coefficient on social loneliness is −1.603 (*p* < 0.01). The effect of PAR on emotional loneliness shows no statistical significance in this group.

**Table 4:** Heterogeneity analysis: grouped regression results by age group.

Variable	Younger-old adults			Older-old adults		
	Overall loneliness	Emotional loneliness	Social loneliness	Overall loneliness	Emotional loneliness	Social loneliness
PAR	0.932^***^	7.834	−1.603^***^	0.456^***^	3.291^***^	−1.469^***^
	(0.363)	(0.452)	(0.289)	(0.221)	(0.377)	(0.183)
Controls	YES	YES	YES	YES	YES	YES
Region	YES	YES	YES	YES	YES	YES
Loglikelihood	−194.45	−151.31	−211.75	−447.33	−454.71	−534.44
Pseudo-*R*^2^	0.581	0.684	0.612	0.608	0.622	0.549
*N*	312	312	312	769	769	769

*, **, *** Indicate significance at the 10, 5, and 1% levels, respectively. The values in parentheses represent standard errors.

Within the older-old adult group, PAR demonstrates significant effects on all three types of loneliness. The coefficient for overall loneliness is 0.456 (*p* < 0.01), for emotional loneliness is 3.291 (*p* < 0.01), and for social loneliness is −1.469 (*p* < 0.01). This age heterogeneity likely stems from systematic differences in social adaptation capacity and emotional dependency structures between age groups. Younger-old adults generally possess better physical mobility and greater willingness for social participation. Consequently, they can more actively utilize the community activities and public spaces provided in resettlement areas, effectively establishing new social connections and alleviating social loneliness. The intergenerational separation and family dispersal caused by PAR may not yet fundamentally impact their emotional support systems.

In contrast, older-old adults often face limitations in physical and social activity capacity. They encounter greater difficulties in environmental adaptation and network reconstruction. Furthermore, their emotional dependencies are more deeply embedded in original kinship and neighborhood relationships. The rupture of these strong-tie networks caused by PAR delivers a more severe impact on their emotional world, resulting in significantly aggravated emotional loneliness. Although community activities in resettlement areas provide some mitigation for their social loneliness, this positive effect is far from sufficient to offset the substantial losses experienced in the emotional dimension.

### 5.3 Endogeneity test

Because the selection of households into relocation may be correlated with unobserved factors—such as household economic capacity, health conditions, or local government implementation preferences—direct estimation may suffer from endogeneity bias, making an instrumental-variable approach necessary. Table 5 first reports the first-stage regression results. The results show a significant positive association between geological disaster risk and participation in PAR (coefficient = 0.094, *p* < 0.01), indicating that villages facing higher geological hazard risk are more likely to be included in the relocation plan. The first-stage F statistic is 28.63 and the Cragg–Donald Wald F statistic is 39.17, both exceeding the conventional threshold of 10, suggesting no serious weak-instrument concern. In addition, the endogeneity tests (insig_2 and atanhrho_12) are both significant, confirming that relocation is endogenous and that the instrumental-variable approach is warranted.

**Table 5:** Estimation results based on the instrumental variable (IV) approach.

Variable	First stage	Second stage		
	PAR	Overall loneliness	Emotional loneliness	Social loneliness
Risk	0.094^***^			
	(0.018)			
PAR		2.882^***^	5.361^***^	−3.625^***^
		(0.509)	(0.239)	(0.375)
Controls	YES	YES	YES	YES
Region	YES	YES	YES	YES
lnsig_2		−1.383^***^	−1.383^***^	−1.383^***^
		(0.022)	(0.022)	(0.022)
atanhrho_12		−0.740^***^	−1.039^***^	−0.749^***^
		(0.233)	(0.220)	(0.219)
*N*	1,081	1,081	1,081	1,081

*, **, *** Indicate significance at the 10, 5, and 1% levels, respectively. The values in parentheses represent standard errors.

In the second-stage regression, the direction and statistical significance of the relationship between relocation and loneliness among older adults remain robust, which enhances the credibility of the findings to some extent. Under the IV specification, the estimated coefficients of PAR on overall loneliness, emotional loneliness, and social loneliness are 2.882 (*p* < 0.01), 5.361 (*p* < 0.01), and −3.625 (*p* < 0.01), respectively. Compared with the baseline results, the absolute magnitudes of the IV estimates are larger. This pattern suggests that, once potential endogeneity in relocation decisions is taken into account, the association between relocation and loneliness may have been underestimated, and the IV approach partially corrects for this bias. That said, IV estimation relies on specific identification assumptions. The IV results should therefore be interpreted as complementary evidence to the baseline regressions, rather than as the sole characterization of the causal effect.

### 5.4 Robustness test

To ensure the reliability of the research findings, this study conducts robustness checks by replacing the dependent variable and excluding a specific subpopulation. Table 6 shows the results.

**Table 6:** Robustness tests of PAR’s impact on loneliness among older adults.

Variable	Change indicators	Exclude samples		
	Overall loneliness	Overall loneliness	Emotional loneliness	Social loneliness
PAR	0.462^**^	0.440^*^	3.394^***^	−1.278^***^
	(0.159)	(0.187)	(0.349)	(0.155)
Controls	YES	YES	YES	YES
Region	YES	YES	YES	YES
Log likelihood	−566.716	−650.69	−535.04	−705.48
Pseudo-*R*^2^	0.555	0.554	0.618	0.508
*N*	1,081	942	942	942

*, **, *** Indicate significance at the 10, 5, and 1% levels, respectively. The values in parentheses represent standard errors.

First, the study integrates the indicators for emotional loneliness and social loneliness to construct a binary loneliness variable. This variable takes a value of 1 if an older adult scores high on either dimension, and 0 otherwise. Re-estimating the model using this new indicator shows a coefficient for PAR of 0.462, which is significant at the 5% level. This result indicates that PAR significantly increases the risk of older adults experiencing a state of loneliness. Second, considering the unique circumstances of older adults living alone, whose loneliness might primarily stem from their family structure rather than PAR, the study re-estimates the models after excluding this group. The results demonstrate that, after excluding older adults living alone, the coefficient for PAR on overall loneliness is 0.440 (*p* < 0.1), on emotional loneliness is 3.394 (*p* < 0.01), and on social loneliness is −1.278 (*p* < 0.01). All coefficients retain their statistical significance and their directions remain consistent with the baseline regression.

The combined results from these two robustness checks confirm that the impact of PAR on loneliness among older adults remains significant. The study’s conclusions demonstrate good robustness.

### 5.5 Mechanism effect analysis

Table 7 presents the test results for the mediating mechanism of social exchange intensity between PAR and loneliness among older adults. Following the mediation effect testing procedure, this study conducts separate tests for overall loneliness, emotional loneliness, and social loneliness.

**Table 7:** Moderation test: buffering effects of follow-up support policies.

Variable	(1)	(2)	(3)	(4)	(5)	(6)	(7)
	Social exchange intensity	Overall loneliness	Overall loneliness	Emotional loneliness	Emotional loneliness	Social loneliness	Social loneliness
PAR	0.623^***^	0.568^***^	0.493^**^	3.592^***^	3.505^***^	−1.486^***^	−1.431^***^
	(0.147)	(0.183)	(0.192)	(0.351)	(0.342)	(0.152)	(0.153)
Social exchange intensity			0.896^***^		0.518^***^		−0.416^***^
			(0.094)		(0.082)		(0.076)
Controls	YES	YES	YES	YES	YES	YES	YES
Region	YES	YES	YES	YES	YES	YES	YES
Loglikelihood		−655.5	−606.2	−616.9	−588.5	−755.4	−736.0
Pseudo *R*^2^	0.534	0.591	0.622	0.634	0.651	0.548	0.560
*N*	1,081	1,081	1,081	1,081	1,081	1,081	1,081

*, **, *** Indicate significance at the 10, 5, and 1% levels, respectively. The values in parentheses represent standard errors.

In the path analysis for overall loneliness, column (1) shows the total effect of PAR on overall loneliness is 0.568 (*p* < 0.01). Column (2) indicates that PAR exerts a significant positive influence on social exchange intensity (*β* = 0.623, *p* < 0.01). After including both PAR and social exchange intensity in column (3), social exchange intensity demonstrate a significant positive influence on loneliness (*β* = 0.896, *p* < 0.01), while the direct effect coefficient of PAR decreases from 0.568 to 0.493 (*p* < 0.05). This result confirms that social exchange intensity play a partial mediating role between PAR and overall loneliness.

In the path analysis for emotional loneliness, column (4) shows the total effect of PAR on emotional loneliness is 3.592 (*p* < 0.01). Column (6) indicates that social exchange intensity exert a significant positive influence on emotional loneliness (*β* = 0.518, *p* < 0.01), and the direct effect coefficient of PAR decreases from 3.592 to 3.505 (*p* < 0.01). This verifies the partial mediating effect of social exchange intensity in the relationship between PAR and emotional loneliness.

In the path analysis for social loneliness, column (7) shows the total effect of PAR on social loneliness is −1.486 (*p* < 0.01). Column (7) shows that social exchange intensity exert a significant negative influence on social loneliness (*β* = −0.416, *p* < 0.01), and the direct effect coefficient of PAR changes from −1.486 to −1.431 (*p* < 0.01). This validates the partial mediating role of social exchange intensity between PAR and social loneliness.

Synthesizing the results in Table 7, social exchange intensity exhibit a significant mediating effect in the relationship between PAR and all three types of loneliness. Hypothesis H4 thus receives support. PAR not only directly influences loneliness among older adults but also exerts an indirect influence on loneliness through the pathway of enhancing social exchange intensity. Notably, the direction of social networks’ influence varies across different loneliness dimensions: it shows a positive influence on emotional loneliness and overall loneliness, but a negative influence on social loneliness.

### 5.6 Moderation effect analysis

To examine the moderating effect of support policies on the relationship between PAR and loneliness among older adults, this study introduces an interaction term between PAR and support policies (PAR × Support policies). This approach assesses the buffering role of external policy support in mitigating the psychological impact of PAR. Table 8 presents the results.

**Table 8:** Moderation test: buffering effects of follow-up support policies.

Variable	(1)	(2)	(3)
	Overall loneliness	Emotional loneliness	Social loneliness
PAR	0.601^**^	3.474^***^	−1.396^***^
	(0.201)	(0.353)	(0.195)
Support policies	0.279	−0.223	0.129
	(0.247)	(0.155)	(0.162)
PAR × Support policies	−0.091	−0.393^**^	−0.361^*^
	(0.265)	(0.193)	(0.191)
Controls	YES	YES	YES
Region	YES	YES	YES
Log likelihood	−653.01	−614.79	−752.544
Pseudo *R*^2^	0.593	0.635	0.550
*N*	1,081	1,081	1,081

*, **, *** Indicate significance at the 10, 5, and 1% levels, respectively. The values in parentheses represent standard errors.

Regarding emotional loneliness, the coefficient for the interaction term is −0.393, which is significantly negative at the 5% level. This result indicates that support policies can effectively mitigate the aggravating effect of PAR on emotional loneliness among older adults. For social loneliness, the coefficient for the interaction term is −0.361, showing a significant negative effect at the 10% level. This finding suggests that support policies also function as a moderator in the relationship between PAR and social loneliness. Strengthened policy support further amplifies PAR’s effect on alleviating social loneliness, thereby promoting the social integration of older adults in their new communities. However, for overall loneliness, the coefficient for the interaction term is −0.091 and fails to reach statistical significance. This non-significance may occur because PAR’s aggravating effect on emotional loneliness and its alleviating effect on social loneliness offset each other at the overall level, resulting in an insignificant moderating effect of support policies.

These findings provide empirical evidence for mitigating the negative psychological impacts of PAR on older adults through policy intervention, supporting research hypothesis H5.

**Figure 2:**
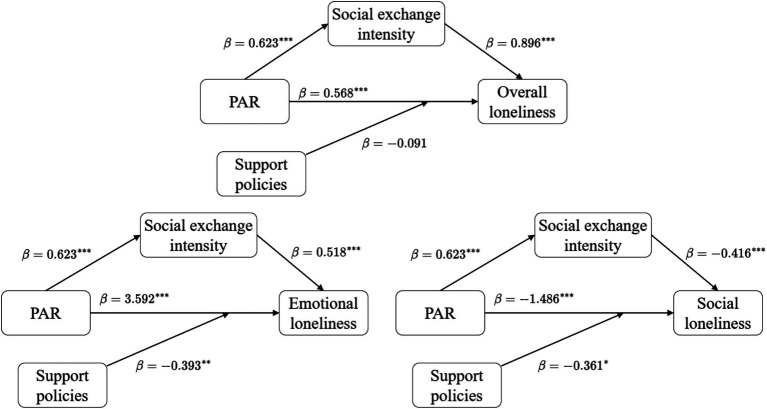
The mediating effect of social exchange intensity and the moderating effect of follow-up support policies.

Figure 2 illustrates the strength of associations between PAR and overall loneliness, emotional loneliness, and social loneliness. It also depicts the mediating and moderating effects of social interaction intensity and supportive policies.

## 6 Conclusion, discussion and policy implications

### 6.1 Conclusions and discussion

Through theoretical and empirical analysis, this study systematically investigates the impact of Poverty Alleviation through Relocation (PAR) on loneliness among older adults and its underlying mechanisms. The analysis yields the following main conclusions. First, PAR is significantly and positively associated with older adults’ overall loneliness, and this association differs markedly across dimensions: it is significantly positive for emotional loneliness but significantly negative for social loneliness. Second, resettlement mode and age moderate the relationship between PAR and loneliness: the positive association between PAR and loneliness is more pronounced among those in centralized resettlement and among older-age groups. Third, social exchange intensity plays a key mediating role in the relationship between PAR and loneliness. By reshaping the structure of social exchange, PAR is indirectly associated with different dimensions of loneliness, revealing a micro-level pathway linking relocation to loneliness. Fourth, post-relocation support policies serve a crucial moderating function in mitigating the psychological impact of PAR. These policies demonstrate significant negative moderating effects in the relationships between PAR and both emotional loneliness and social loneliness.

Based on the above conclusions, this study further develops the following discussion.

First, the impact of PAR on loneliness among older adults shows a divergence between the “emotional” and “social” dimensions, reflecting its differential effects on various aspects of loneliness. While PAR severs the strong-tie networks based on kinship and geography that older adults previously relied on, it also creates conditions for establishing new weak-tie networks within the community. The former leads to a rupture in emotional support systems, aggravating emotional loneliness; the latter, by increasing social opportunities and participation in public activities, alleviates social loneliness to some extent. This suggests that assessments of psychological adaptation in older adults should move beyond overall feelings and deeply examine different dimensions of psychological need.

Second, the impact of centralized resettlement mode on loneliness is more pronounced, while the effect of dispersed resettlement is relatively limited. This highlights the critical role of spatial distance and social structure continuity in the psychological adaptation of older adults. Centralized resettlement often involves long-distance migration and a thorough reorganization of community structures, leading to a severe loss of original social capital. In contrast, dispersed resettlement, due to geographical proximity, allows for the partial preservation of social networks, resulting in a lesser psychological impact. Older-old adults, facing declining physiological function, weakened social roles, and reduced environmental adaptability, find it more difficult to rebuild emotional connections after their social networks fracture. This identifies them as a vulnerable group requiring particular attention in PAR initiatives.

Third, social exchange intensity plays a compound mediating role in the effect of relocation on loneliness, highlighting its dual nature in coping with the psychological shocks induced by PAR: it may alleviate social loneliness by creating new opportunities for social interaction, yet it may be less effective in reducing emotional loneliness because it cannot fully substitute for pre-existing intimate relationships. This finding provides a more nuanced micro-level explanation of the mechanisms through which PAR affects mental health.

Finally, the significant moderating effect of support policies demonstrates that external institutional support can effectively mitigate the psychological stress induced by PAR. By providing emotional care, organizing community activities, and enhancing social participation, policy support partially compensates for the deficiency of informal support, offering a buffer for older adults adapting to their new environment. This empirically verifies the feasibility and importance of policy intervention in promoting the psychological integration of populations undergoing PAR.

### 6.2 Policy implications

Based on the findings of this study, we propose the following recommendations to systematically mitigate the negative impact of PAR on the psychological wellbeing of older adults and enhance the comprehensive benefits of the policy.

First, policymakers should establish differentiated psychological support systems. Policy formulation must fully consider the multidimensional nature of loneliness and implement targeted interventions for emotional loneliness and social loneliness. The study also confirms that PAR affects older adults’ emotional support and social interactions. Therefore, to address emotional loneliness, it is important to strengthen older adults’ emotional support systems by establishing regular visitation arrangements, promoting intergenerational communication, and—where feasible—moderately preserving their original community networks. To address social loneliness, efforts should focus on improving community public spaces, organizing interest-group activities, and providing opportunities for community participation, thereby helping older adults integrate more quickly into resettlement communities and form stable social ties.

Second, innovate mechanisms for rebuilding older adults’ social interactions. The study shows clear differences across resettlement modes in their effectiveness in alleviating loneliness. Accordingly, policy interventions should adopt a mode-specific approach, using resettlement mode as the entry point for differentiated measures. For centralized resettlement communities, building on existing advantages in public spaces and organized activities, greater emphasis should be placed on strengthening emotional support systems—for example, by establishing mutual-aid groups, long-term companionship programs, and priority care lists to provide more targeted support for emotional loneliness. For dispersed resettlement groups, measures such as a community liaison system, designated contact points, regular home visits, and transportation subsidies are needed to proactively repair disruptions in social-network reconstruction and prevent prolonged social isolation from evolving into persistent loneliness.

Third, governments need to improve targeted support policies for special populations. Older-old adults face a higher risk of emotional loneliness after relocation, whereas younger-old adults can, to some extent, benefit from increased social opportunities in the community. This suggests that post-relocation community building should not adopt a one-size-fits-all approach. For older-old adults, greater emphasis should be placed on emotional companionship, home-visit programs, and continuous care to compensate for the loss of intimate support. For younger-old adults, initiatives such as volunteering and participation in community governance can further activate their potential for social engagement and maximize the social-integration benefits brought by relocation. For economically disadvantaged families, providing social participation subsidies can lower the financial barriers to engaging in community activities.

Finally, communities should advance service-integrated community governance models. Integrating mental health services organically into routine community work systems can achieve normalization and institutionalization of psychological services for special groups. Simultaneously, strengthening mental health training for community staff will enhance their ability to identify and address psychological issues among older adults, ultimately building a comprehensive psychological wellbeing safeguard system.

## 7 Research limitations and future prospects

Although this study reveals the impact effects of PAR on loneliness among older adults and its underlying mechanisms through theoretical and empirical analysis, certain limitations remain. On the one hand, regarding measurement, this study adopts single-item measures for the three dimensions of loneliness due to respondents’ limited educational attainment and fieldwork feasibility constraints. Although this approach is consistent with the practical realities of surveying hard-to-reach populations and the selected items have reasonable face validity and theoretical grounding, single-item measures may not fully capture the complexity of loneliness as an affective state. Where feasible, future research should employ validated multidimensional instruments (e.g., established loneliness scales) to improve measurement precision. On the other hand, this study is based on cross-sectional data. Although we establish a temporal ordering in which relocation precedes the measurement of the mediator and outcomes, it remains difficult to completely rule out the influence of unobserved factors that may jointly affect the results. Even with an instrumental-variable strategy, endogeneity can only be mitigated to some extent. Future work using panel follow-up data or natural-experimental designs would be better suited to validating the dynamic and causal effects of relocation. Expanding such research to more diverse regional and cultural backgrounds would also allow for valuable comparative studies. Furthermore, subsequent studies could further explore the potential role of factors such as digital technology use and changes in intergenerational support in alleviating loneliness among older adults undergoing relocation.

## Data Availability

The raw data supporting the conclusions of this article will be made available by the authors, without undue reservation.
